# Reproductive Cycle of the Seagrass *Zostera noltei* in the Ria de Aveiro Lagoon

**DOI:** 10.3390/plants10112286

**Published:** 2021-10-26

**Authors:** Manuel Ankel, Marcos Rubal, Puri Veiga, Leandro Sampaio, Laura Guerrero-Meseguer

**Affiliations:** 1 Interdisciplinary Centre of Marine and Environmental Research (CIIMAR), Novo Edifício do Terminal de Cruzeiros do Porto de Leixões, Avenida General Norton de Matos, 4450-208 Matosinhos, Portugal; marcos.garcia@fc.up.pt (M.R.); puri.sanchez@fc.up.pt (P.V.); leandro.sampaio@fc.up.pt (L.S.); laura.meseguer@fc.up.pt (L.G.-M.); 2 Department of Biology, Faculty of Sciences, University of Porto, Rua do Campo Alegre s/n, 4169-007 Porto, Portugal

**Keywords:** sexual reproduction, flowering, conservation, seeds, germination

## Abstract

Sexual reproduction in seagrasses is essential to increase their resilience towards environmental stressors, but its phenology is still unknown in some regions, limiting our knowledge about the recovery capacity of these ecosystems. In this study, the flowering effort, reproductive phenology, seed production and ability of germination of *Zostera noltei* was studied for the first time in the Ria de Aveiro lagoon, Portugal. Flowering of *Z. noltei* in the Ria de Aveiro lasts from June to November, reaching a peak between July and August. All the meadows showed similar flowering effort and phenology over time. Comparing with other European populations, the flowering effort of *Z. noltei* in Ria de Aveiro lasted for a longer period, which could be related with the milder temperatures in summer and autumn and the great anthropogenic stress to which the meadows are subjected in the lagoon. The number of seeds produced and their ability of germination were similar among meadows and sampling periods, reaching levels similar to those of other European regions. Nevertheless, future studies are needed to determine the fate of the produced seeds in the field to have a better understanding about the natural recovery capacity of the species.

## 1. Introduction

Seagrasses are marine angiosperms that form extensive meadows worldwide, hosting a large number of aquatic organisms [1]. Since seagrass meadows support a high biodiversity and provide many ecological services, they are considered to be among the most important ecosystems worldwide [2]. Despite this, a global decline in seagrasses with an accelerating rate of loss has been reported since the 1990s [3]. Anthropogenic stressors, such as poor water quality, coastal development and dredging, have been identified as main drivers for this seagrass decline [4], but extreme weather events derived from global warming are also negatively affecting seagrasses and their sexual reproduction [5,6,7].

In general, the main growth pattern of seagrasses is through asexual cloning of their rhizomes, but as angiosperms, they can reproduce sexually through the formation of flowers, fruits and seeds [2]. Seedling recruitment enhances their genetic diversity and, in consequence, strengthens the resistance and resilience of the seagrass meadows towards environmental stressors [8,9]. Seagrasses follow two strategies when reproducing sexually: the dispersal of seeds by the sea surface and the formation of seedbanks by accumulation of dormant seeds in the sediment [10,11]. For instance, the dwarf eelgrass *Zostera noltei* (Hornemann) produces non-dormant seeds and forms seedbanks in the sediment that can be both annual and persistent [12,13]. The existence of a persistent seedbank guarantees the survival of the seagrass meadows [14], facilitating their recovery after negative impacts [15].

*Zostera noltei* often occurs in sheltered environments such as lagoons and estuaries. This seagrass mainly grows on muddy and sandy sediments on intertidal areas, forming extensive beds [16]. *Zostera noltei* adapts to a wide range of environmental conditions (i.e., different sediment types, nutrient levels, tidal ranges or current velocities), which is reflected in its plasticity on morphological, physiological and population levels [17,18]. The timing of sexual reproduction in *Z. noltei* differs between latitudes. In southern European populations, sexual reproduction usually starts in March/April and lasts until autumn (October/November) [19], whereas at higher latitudes it starts later at the end of June [12]. Sexual reproduction in *Z. noltei* also differs when exposed to different environmental conditions: flowering is enhanced in areas exposed to environmental stressors such as increased hydrodynamics and organic matter enrichment, whereas stable and sheltered areas lead to reduced flowering effort [20,21]. Thus, flowering seems to be variable in this species and influenced by many environmental factors.

The Ria de Aveiro lagoon holds the second largest *Z. noltei* population in Portugal [22], covering around 2.3 km^2^ in 2014 [23]. In the last decade, some studies have addressed the vegetative growth of *Z. noltei* under different environmental conditions [24,25], as well as its role as blue carbon sink in the Ria de Aveiro lagoon [23]. Nevertheless, the reproductive capacity of the species has never been taken in consideration when evaluating its conservation status in the lagoon. A recent study suggests that there is a relationship between the reproductive effort of this species and the content of organic matter and silt in the sediment [21]. However, there are no baseline data on the phenology and germination ability of *Z. noltei* in Ria de Aveiro which allows us to compare the reproductive capacity of the species over time. This lack of knowledge limits our understanding of the natural colonisation capacity of this seagrass in the area and, in consequence, the understanding on how future disturbances, such as climate change, could affect its sexual reproduction.

The aim of this study was to describe for the first time the phenology and reproductive capacity of *Z. noltei* in the Ria de Aveiro lagoon. To achieve this goal, we monitored the flowering effort, reproductive phenology and germination ability of four reproductive meadows throughout the flowering period of the species.

## 2. Materials and Methods

### 2.1. Study Area

**Figure 1:**
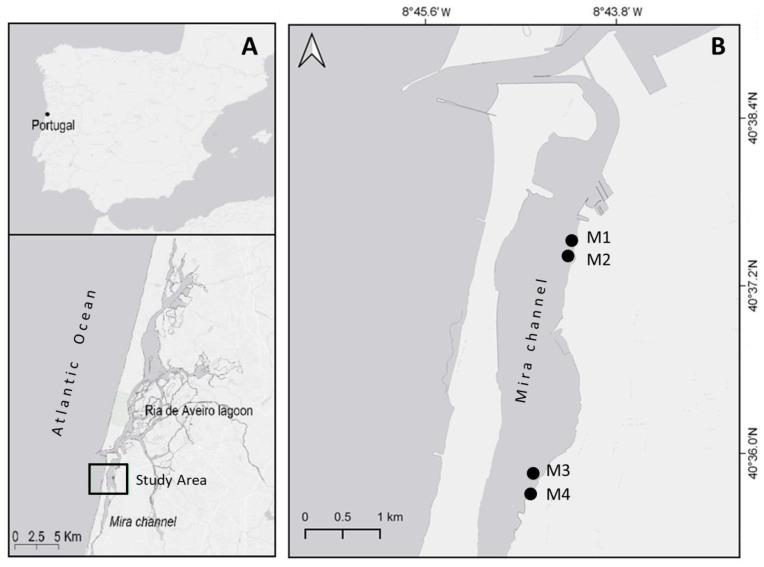
Study area (**A**) and location of the four studied *Z. noltei* meadows (M1-M4) in the Mira channel, Ria de Aveiro (**B**).

To study the sexual reproduction cycle of *Z. noltei* in Ria de Aveiro (40°38′ N, 8°45′ W), four monospecific seagrass meadows that showed reproductive capacity were visited along the Mira channel while their flowering persisted in the lagoon. The Mira channel is a shallow arm with 20 km in length [26] which shows characteristics of a seasonally poikilohaline estuary with salinity ranges from 0 to 35 psu (practical salinity unit) [27]. Nearly one-fifth of the tidal water volume diverts into the Mira channel, whereas at its upper end, a small network of lagoons and streams constantly delivers freshwater. To assess spatial variability in sexual reproduction, the sampling was carried out using two spatial scales, meters and kilometers. Thus, meadows M1 and M2 were about 200 m apart, the same distance between M3 and M4, and M1–M2 were 3 km away from M3–M4 (Figure 1).

In this channel, all the studied *Z. noltei* meadows had a similar seawater temperature before (May), during (August) and after (December) the flowering period (27.94 ± 0.65 °C, 25.24 ± 0.85 °C and 13.75 ± 0.14 °C, respectively; Table S3), but salinity, grain size and sediment organic matter were variable over time. Thus, those three parameters were recorded in each meadow over the study period to analyse differences among meadows.

Seawater salinity was recorded at two randomly selected points at each sampling date and meadow, using a multi sonde (HQ 40 d, Hach, Düsseldorf, Germany). To analyse organic matter content and sediment grain size, sediment corers (n = 2, 5.5 cm diameter, 7 cm of length) were taken before, during and after the sexual reproduction period. Sediment corers were dried for 72 h at 60 °C to measure the organic matter content of the sediment (OM) by loss on ignition in 1 g of sediment (450 °C, 4.5 h). Then, sediment was separated into seven sizes using a mechanical sieve shaker (CISA# SIEVING TECHNOLOGIES BA 200 N; t = 20 min, amplitude = 1.2 mm), and subsequently classified following the Wentworth scale [28]: fine gravel (2–4 mm), very coarse sand (1–2 mm), coarse sand (0.5–1 mm), medium sand (0.25–0.5 mm), fine sand (0.125–0.25 mm), very fine sand (0.063–0.125 mm) and silt and clay (<0.063 mm).

### 2.2. Flowering Effort and Reproductive Phenology

To study the flowering effort and the reproductive phenology of *Z. noltei* in the Ria de Aveiro lagoon, seagrass corers (n = 4; 9 cm of diameter, 6.5 cm of length) were randomly collected at five dates (June, July, August, September and November) in each meadow as the flowering period persisted. At the laboratory, seagrass corers were cleaned from fauna and sediment with artificial seawater (30 psu). Then, the sexual spathes contained in each corer were counted for recording the flowering effort.

**Figure 2:**
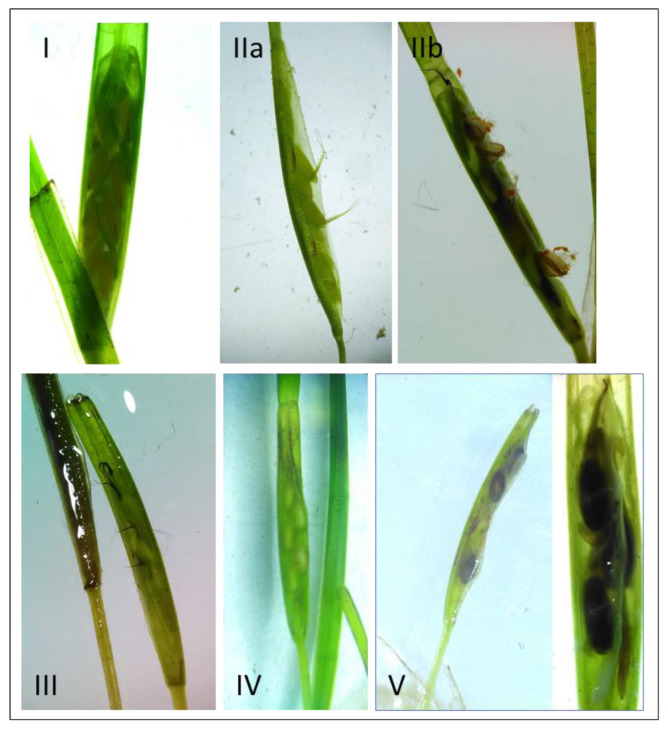
Morphology of the five sexual reproductive stages (**I**–**V**) determined to study the reproductive phenology of the *Z. noltei* meadows at the Ria de Aveiro lagoon.

The reproductive phenology of *Z. noltei* in Ria de Aveiro was assessed by analysing the spathes under a dissecting microscope and assigning them a stage of development based on their morphological characteristics. In this way, five different stages of sexual reproduction were defined (Table 1, Figure 2). Stages I, II and III corresponded to the period of flowering formation, while stages IV and V coincided with the period of seed formation and maturation. Broken and necrotic spathes were quantified as abortions.

The total number of spathes collected in each reproductive stage during the study period was calculated to describe the general reproductive phenology of *Z. noltei* in the Ria de Aveiro lagoon. To test differences among meadows in the reproductive phenology, the percentage of spathes in each reproductive stage was estimated by meadow and date.

### 2.3. Germination Ability

To test the germination ability of the *Z. noltei* meadows, all collected sexual spathes, regardless of their stage of sexual reproduction, were separated per meadow and sampling date and cultured in aquaria (10 cm of diameter, filled with 600 mL artificial seawater) until mature seeds were obtained to apply for germination tests.

The salinity in each aquarium was adjusted every 72 h to the mean seawater salinity obtained in each *Z. noltei* meadow in the corresponding sampling date (Table S1). Aquaria were aerated (Air 550 R Plus, Sera GmbH, Heinsberg, Germany; O_2_ flux > 5 mg/L) and maintained at room temperature (from 18.35 ± 0.20 °C to 23.66 ± 0.12 °C) under natural photoperiod (from 14:10 to 12:12 light/dark hours, from July 2019 to March 2020). Every week, mature seeds, with hard, dark brown seed coat, were collected, counted and stored at salinities above 33 psu in 50 mL tubes filled with artificial seawater to avoid natural seed germination before the test. The seeds were kept stored in the tubes until March 2020, when the germination tests were carried out, coinciding with the beginning of spring and the natural germination period of the species [29]. Spathes that suffered senescence were removed from the aquaria to avoid their decomposition. To prevent overgrowth of epiphytes, artificial seawater was renewed, and the aquaria and aeration system were cleaned once a week. Seed production was expressed as the number of seeds per spathe.

Germination tests were conducted by exposing mature seeds to a low salinity shock to induce germination [12]. Seeds were introduced into aerated tubes (50 mL) filled with 20 mL of 1 psu artificial seawater and 20 mL of agar (5% at 1 psu) as neutral substrate and placed inside an environmental chamber with controlled temperature and photoperiod (25.00 ± 1.026 °C, 12:12 light/dark hours, respectively). Then, the number of germinated seeds (with broken seed coat and visible cotyledon) was recorded every week for four weeks to calculate the ability of germination. The germination ability was expressed as percentage of germinated seeds per meadow. Seeds from M4 were not considered for this experiment since they were not mature or had died during the storage. Aeration of the tubes was provided as described above. Salinity was monitored weekly in four randomly selected tubes and adjusted when 3 psu were surpassed.

### 2.4. Data Analyses

To test if the environmental conditions (salinity, OM and sediment grain size), flowering effort and reproductive phenology differed among meadows and through the reproductive period of *Z. noltei*, two-way ANOVAs were done using *meadow* as fixed factor with four levels (M1, M2, M3 and M4) and *time* as random factor with three levels for environmental conditions (before, during and after sexual reproduction period) and four levels for flowering effort and reproductive phenology (July, August, September and November). Prior to the analyses, data were checked for normality and homogeneity of variances and transformed when necessary to fulfil ANOVA assumptions. If transformed data did not meet the assumptions, the significance level (*α*) was lowered to 0.01 [30].

To test if seed production was different among meadows, *t*-tests were separately applied. Fisher’s exact test of independence was used to explore significant differences on germination ability among meadows.

All the tests were performed with the statistical software R Version 4.0.0 [31]. All the results throughout this article have been expressed as mean ± standard error (SE).

## 3. Results

### 3.1. Spatio-Temporal Variability in Environmental Conditions

**Figure 3:**
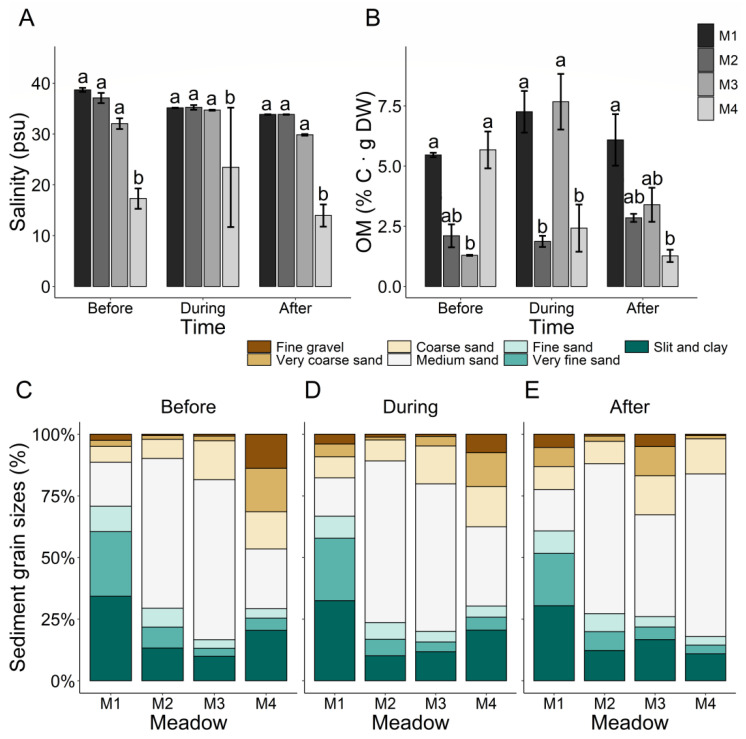
Salinity (**A**), organic matter content (**B**) and percentages of each sediment grain size (**C**–**E**) (mean ± SE; n = 2) amidst the four studied *Z. noltei* meadows (M1–M4) before, during and after the flowering period. Letters above error bars indicate significant differences among meadows.

Seawater salinity was significantly lower in M4 than in the remaining meadows before, during and after the flowering period (Figure 3A, Table 2). The organic matter content was very variable in space and time in the *Z. noltei* meadows (Figure 3B, Table 2).

The sediment grain sizes also revealed a high spatio-temporal variability in the *Z. noltei* meadows, except in coarse and very fine sand content, which only showed differences among meadows (Table 2). The percentages of coarse sand in M1 and M2 were significantly lower than in M3 and M4 along the flowering period (Figure 3C–E). Regarding very fine sand, M1 and M2 showed higher values than the other meadows throughout the flowering period (Figure 3C–E).

### 3.2. General Reproductive Phenology of Z. noltei in the Ria de Aveiro Lagoon

**Figure 4:**
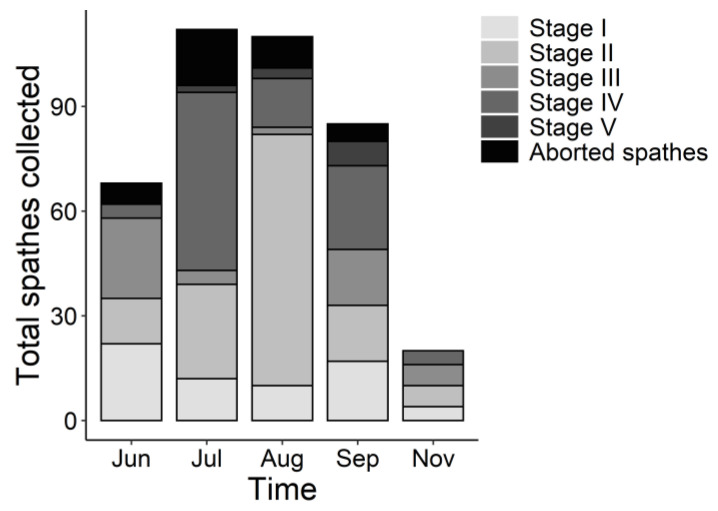
General reproductive phenology of the studied *Z. noltei* meadows of the Ria de Aveiro lagoon.

The flowering period of *Z. noltei* in the Ria de Aveiro lagoon lasted approximately from June to November 2020. Since spathes with seeds were found in June and there were still spathes with flowers in formation in November, the flowering of *Z. noltei* in the lagoon could begin a little before June and end sometime after November. In general, the number of spathes reached a peak in July and decreased in the following months (Figure 4). The number of Stage I spathes was similarly maintained over the flowering period, while Stage II spathes reached maximal values in August (Figure 4). The number of Stage III spathes peaked in June and September, while Stage IV reached the highest values in July and Stage V values increased slightly from July to September (Figure 4). Moreover, the number of aborted spathes peaked in July and declined in the following months (Figure 4).

### 3.3. Spatio-Temporal Variability in the Flowering Effort and Reproductive Phenology

**Figure 5:**
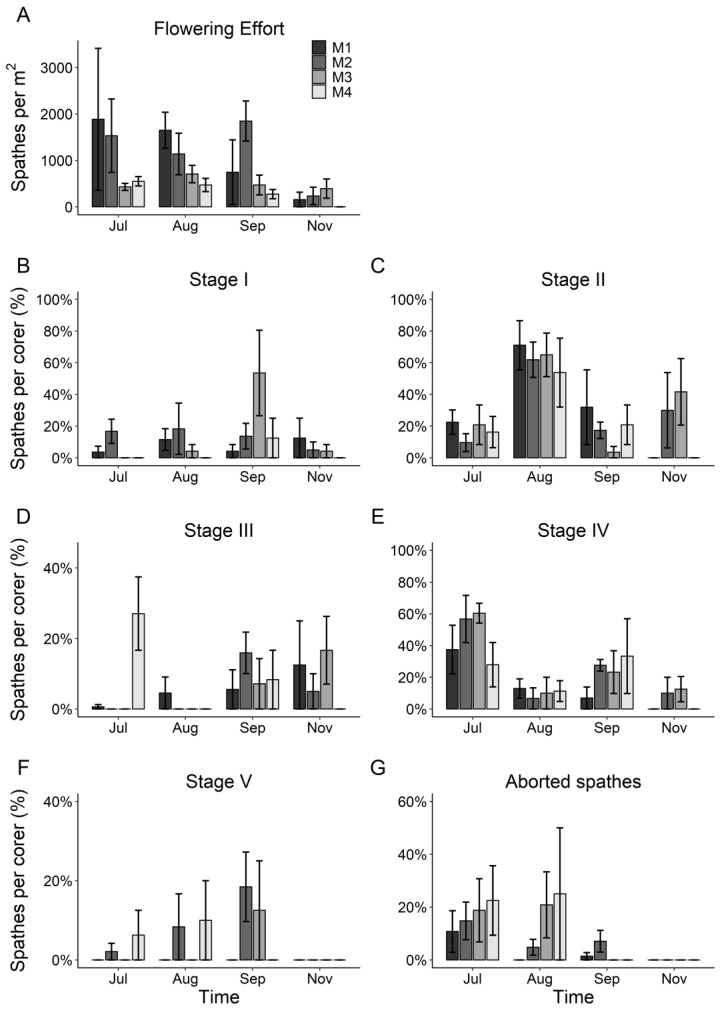
Flowering effort (**A**), percentages of spathes in each reproductive stage (**B**–**F**) and percentage of aborted spathes (**G**) over time (mean ± SE, n = 4).

Flowering effort did not significantly differ among meadows and time (Table 3; Figure 5A), showing an average of 781 ± 157 spathes · m^−2^ per *Z. noltei* meadow. Similarly, there were no significant differences among meadows and time regarding the aborted spathes and the percentages of spathes in stages I, III and V (Table 3; Figure 5B,D,F,G). However, the percentage of spathes in stages II and IV were significantly variable over time (Table 3; Figure 5C,E).

### 3.4. Germination Ability

A total of 158 seeds were obtained from the cultured spathes. Seed production did not differ significantly among meadows (M1–M2, *t* = 0.443, *p* = 0.669; M1–M3, *t* = 0.482, *p* = 0.643 and M2–M3, *t* = 0.786, *p* = 0.455), averaging 0.422 ± 0.109 seeds per collected spathe. Amid these seeds, 51.26% (81 seeds) were fully matured and used for the germination test (Table S2). Similarly, germination capacity did not show significant differences among meadows (Table 4), germinating 33.73% of mature seeds. The average germination time of the seeds formed in M1, M2 and M3 was 1.000 ± 0.001, 1.389 ± 0.164 and 2.200 ± 0.490, respectively.

## 4. Discussion

This study shows the first data on reproductive phenology of *Zostera noltei* in the Ria de Aveiro lagoon. The flowering period of *Z. noltei* in Ria de Aveiro started around June and lasted until near November, peaking between July and August. Flowering effort and reproductive phenology were similar among meadows and over time, suggesting that all meadows evolved similarly over the flowering period. The proportion of newly formed spathes (Stage I), apparently fertilised spathes (Stage III) and spathes with mature seeds (Stage V) was constant over time. In contrast, the percentages of spathes in Stage II and Stage IV differed over time, which suggests that the development of these stages could be prompted under certain environmental conditions in *Z. noltei*. The production of seeds and the capacity and time of germination of the cultured spathes did not differ among meadows, showing 34% of germination ability. These results suggest that *Z. noltei* meadows of the Ria de Aveiro lagoon can produce seedlings similarly and with a potential of reproduction comparable to other European populations.

Flowering effort of *Z. noltei* in Ria de Aveiro did not show spatio-temporal differences, ranging from 0−157 to a maximum of 6445 spathes m^−2^. This range is slightly higher than that found for *Z. noltei* in the south of Portugal [19,32] and in the Mediterranean [33,34] but lower than in other European areas further north to Ria the Aveiro [35]. Latitudinal differences in the flowering effort of *Z. noltei* appear to be due to the fact that the southern populations were more stable than the Northern Europe populations and do not have to recolonise the environment each year in the same manner as those further north [36]. On the other hand, the timing of flowering in the *Z. noltei* meadows of Ria de Aveiro, which persisted from June to November, was similar to that reported in the south of Portugal [19] and for European areas further north from our study area [13,35] but longer than in the Mediterranean [33,34]. Therefore, these data could suggest that the timing of flowering in *Z. noltei* is strongly linked to seawater temperature, starting when temperatures rise in the area and persisting as long as warm temperatures remain, but are not too extreme for the development of spathes, as reported in the Mediterranean [34]. However, in Ria de Aveiro, factors other than temperature could have influenced the seagrass reproductive effort since flowering in *Z. noltei* continued even though seawater temperature dropped to 14 °C in November.

Greater reproductive efforts and longer flowering periods in seagrass meadows are also indicators of stress due to disturbances in the area, which can occur naturally or as result of anthropogenic activities [37]. Seagrasses react with increased flowering under stressful situations as an adaptation strategy to ensure the recolonisation of impacted areas through the formation of seedbanks [15,38]. Since, the Ria de Aveiro lagoon is under high anthropogenic influence, numerous stressors could have influenced the flowering of *Z. noltei* in our study, explaining the similarity between the flowering effort observed in Ria de Aveiro and other stressed *Z. noltei* meadows of Portugal. For instance, shellfish and bait harvesting can extend the reproductive period and induce greater production of spathes in *Z. noltei* meadows [19] and we found traces of this activity along the entire Mira channel (Figure S1). Furthermore, a longer flowering period in *Z. noltei* can also be associated with sandy sediments [19,35]. Ria de Aveiro has historically been subjected to major hydromorphological alterations which resulted in changes from finer to sandier or coarser sediments within the lagoon [39]. Moreover, in the year of our study, dredging activities were initiated upstream of the Mira channel, resulting in the mobilisation of fine gravel and very coarse sand along the meadows (see Figure 3C–E). On the other hand, the flowering effort of human-dominated *Z. noltei* meadows of Ria de Aveiro is strongly linked to silty sediments which carry high contents of organic matter [21]. Although in our study the grain size and organic matter content of the sediments were variable, relatively high values (above 2% · g DW) were found in all *Z. noltei* meadows throughout the study period. Mineralisation of organic matter is the main process that supplies inorganic nitrogen and phosphorus to the porewater of marine sediments [2,40]. Consequently, a high content of organic matter in the sediments of Ria de Aveiro during the flowering period could have been responsible for a higher nutrient uptake in *Z. noltei* meadows, extending the period of sexual spathes formation in the lagoon.

The continuous formation of Stage I spathes during the study period supports the fact that sexual spathes can be continually developed in Ria de Aveiro throughout the summer and part of the autumn [19]. Moreover, Stage II corresponded to the moment when the spathes opened to expose the female and male organs to facilitate pollination, and the percentage of Stage II spathes reached a peak in August, suggesting that, in *Z. noltei*, longer exposure to high temperatures could be responsible for the opening of spathes. Since pollination results in the formation of seeds, the high proportion of Stage II spathes observed in August could be the origin of the great number of Stage IV spathes in September, which already carry immature, green seeds in their interior. Following this assumption, the peak of Stage IV spathes in July could be explained by the first cohort of spathes in Ria de Aveiro.

Other environmental stressors in Ria de Aveiro could have also influenced flowering in *Z. noltei*. Desiccation of the seagrass meadows could be one additional factor since *Z. noltei* in Ria de Aveiro is exposed daily for several hours to high temperature and light incidence during low tide. In addition, although the four studied *Z. noltei* meadows had similar values of temperature during the flowering period, they had different ranges of seawater salinity. Stormwater runoffs can transport freshwater from the storm drain outlets to the Mira channel, reducing salinity in certain areas [26,41]. This effect was noticed in M4 in our study, which showed lower salinities than in the rest of the meadows. However, reproductive effort and phenology in this meadow were similar to others that were exposed to salinities above 30 psu. Therefore, salinity seems to have no effect on the flowering in *Z. noltei* in Ria de Aveiro. Nevertheless, it should be noted that the spathes collected in this meadow produced less mature seeds in the laboratory than the others, which suggests that salinity could negatively affect the formation of seeds in *Z. noltei*.

Cultured spathes did not show significant differences among meadows and sampling dates in the production of mature seeds. The production of seeds per spathe reported here (0.42 seeds · collected spathe) was within the range of other studies (around 0.2 to 0.7 seeds per spathe) [12,29]. Similarly, the germination potential of the *Z. noltei* seeds from Ria de Aveiro (34%) was comparable to those reported in other studies that induced germination at similar environmental conditions found in our experiment (30–42%) [12,13,29]. The interplay between environmental factors that enhance germination in *Z. noltei* is still poorly understood and, therefore, more studies are needed to improve the in vitro germination and especially the survival rates of the seagrass seedlings. This knowledge could help to understand which conditions benefit the natural colonisation of this species, and at the same time improve the management for its conservation. In addition, refining the culture of *Z. noltei* seedlings in laboratory would allow us to promote the environmental restoration of their meadows through efficient seeding with a higher survival rate of transplants and an accelerated rate of recovery [42].

This study presents the first baseline data addressing the sexual reproduction of *Z. noltei* in the Ria de Aveiro lagoon. Flowering effort of *Z. noltei* in Ria de Aveiro was similar among meadows and resulted in the successful production of seeds. The flowering period was longer than in other further north European areas and in the Mediterranean, probably due to long lasting mild temperatures in summer and autumn in the Ria de Aveiro and because this area is subjected to several anthropogenic activities and local environmental stressors. The occurrence of different reproductive stages in *Z. noltei* followed a temporal pattern in which a peak in the pollination stage was followed by a peak in the seed formation stage. *Z. noltei* in Ria de Aveiro produced seeds with similar germination ability compared to other populations. Future studies in Ria de Aveiro are needed to investigate factors that influence flowering, the in situ germination of seeds as well as the existence of seedbanks. Furthermore, studies in different parts of Ria de Aveiro are needed for the comparison of the reproductive effort and phenology among different sites of the lagoon, and also for assessing the connectivity of the *Z. noltei* meadows. A complete understanding of the sexual reproduction will help future decision makers to conserve and restore this species in the lagoon.

## Figures and Tables

**Table 1:** Description of the five sexual reproductive stages used to study the reproductive phenology of *Z. noltei* in Ria de Aveiro. The morphology of each reproductive stage is shown in Figure 2.

Period	Stage	Description
Flowering	I	Yellow-green spathe, sheath closed; pistils and stamina are visible, aligned onto the stem
	II	Pistils (IIa) and/or stamina erected (IIb); styles and stigma and/or anthers are outside the sheath
	III	Stigma brown, start to depart from spathe; often with stamina already detached from the spathe
Seed formation	IV	Green spathe with immature seeds; sheath closed
	V	Green/brown spathe, dark brown seeds are visible, sheath open

**Table 2:** Summary of the results obtained in the two-way ANOVA analyses applied for the environmental descriptors. Results are expressed by the *F*-values and associated *p*-values (in parenthesis). Significant results are in bold. Asterisks above the variables indicate that significance level (α) was lowered to 0.01 because data did not fulfil the ANOVA assumptions.

Variable	Meadow (df = 3)	Time (df = 2)	Meadow × Time (df = 6)
Salinity	**16.59 (<0.001)**	1.632 (0.236)	0.474 (0.815)
OM	18.87 (<0.001)	4.820 (0.029)	**10.33 (<0.001)**
Fine gravel	17.42 (<0.001)	0.211 (0.812)	**13.38 (<0.001)**
Very coarse sand	12.50 (<0.001)	0.368 (0.699)	**11.79 (<0.001)**
Coarse sand	**25.76 (<0.001)**	0.516 (0.609)	0.460 (0.830)
Medium sand *	42.99 (<0.001)	0.677 (0.527)	**7.480 (0.002)**
Fine sand	196.5 (<0.001)	0.808 (0.467)	**3.506 (0.031)**
Very fine sand *	**446.1 (<0.001)**	3.187 (0.078)	4.176 (0.017)
Slit and clay	52.27 (<0.001)	0.488 (0.626)	**5.631 (0.005)**

**Table 3:** Results obtained in the two-way ANOVA analyses for FE and stages of sexual reproduction. Results are expressed as *F*-values and associated *p*-values (in parenthesis). Significant results are in bold. The significance level in all tests was α = 0.01.

Variable	Meadow (df = 3)	Time (df = 3)	Meadow × Time (df = 9)
Flowering effort	2.853 (0.047)	2.495 (0.071)	0.708 (0.699)
Stage I	1.274 (0.294)	2.290 (0.090)	1.782 (0.097)
Stage II	0.408 (0.748)	**10.37 (>0.001)**	1.010 (0.446)
Stage III	0.287 (0.834)	1.470 (0.235)	2.282 (0.032)
Stage IV	1.150 (0.339)	**10.92 (>0.001)**	0.807 (0.612)
Stage V	1.266 (0.297)	1.595 (0.203)	1.047 (0.418)
Aborted spathes	0.774 (0.514)	3.375 (0.026)	0.534 (0.842)

**Table 4:** Percentage of germinated and no germinated seeds. Comparison among meadows was done through Fisher’s exact test of independence.

Meadow	Germinated (%)	No Germinated (%)
M1	24	76
M2	43.9	56.1
M3	33.3	66.7
Fisher’s exact test of independence	2.69	*p* = 0.253

## Supplementary Materials

The following are available online at https://www.mdpi.com/article/10.3390/plants10112286/s1, Figure S1: Area impacted by shellfish and bait harvesting in the Mira channel of the Ria de Aveiro, Table S1: Seawater salinity (psu) of the aquaria where the sexual spathes were cultured (mean ± SE) of the salinities measured in each meadow, Table S2: Number of mature seeds collected from aquaria per meadow and date of seed production, Table S3: Seawater temperatures (mean ± SE) among the four studied *Z. noltei* meadows before (May), during (August) and after (December) sexual reproduction period.
