# Supplementary material for: Reproductive Cycle of the Seagrass Zostera noltei in the Ria de Aveiro Lagoon
Source: Plants (Basel). 2021 Oct 26;10(11):2286. doi: 10.3390/plants10112286 (PMC8621667; doi:10.3390/plants10112286)
Supplement: Supplementary file 1 [file plants-10-02286-s001.zip › Table S1.pdf]

**Table S1** Seawater salinity (psu) of the aquaria where the sexual spathes were cultured (mean ± SE) of the salinities measured in each meadow.

| | June | July | August | September | November |
|---|---|---|---|---|---|
| **M1** | 36.10 ± 0.200 | 34.00 ± 0.000 | 35.15 ± 0.050 | 34.55 ± 0.150 | 33.85 ± 0.050 |
| **M2** | 35.15 ± 0.250 | 35.00 ± 0.000 | 35.25 ± 0.450 | 33.90 ± 0.600 | 33.85 ± 0.050 |
| **M3** | 31.90 ± 0.100 | 32.90 ± 1.100 | 34.70 ± 0.100 | 32.20 ± 0.200 | 29.85 ± 0.150 |
| **M4** | 28.70 ± 0.600 | 24.07 ± 9.030 | 23.45 ±11.750 | 17.37 ± 4.130 | 13.67 ± 2.490 |
