# Supplementary material for: Reproductive Cycle of the Seagrass Zostera noltei in the Ria de Aveiro Lagoon
Source: Plants (Basel). 2021 Oct 26;10(11):2286. doi: 10.3390/plants10112286 (PMC8621667; doi:10.3390/plants10112286)
Supplement: Supplementary file 1 [file plants-10-02286-s001.zip › Table S2.pdf]

Table S2. Number of mature seeds collected from aquaria per meadow and date of seed production.

	**June**	**July**	**August**	**September**	**November**	**Total**
**M1**	1	10	12	2	0	**25**
**M2**	4	0	8	28	1	**41**
**M3**	3	0	3	7	2	**15**
**M4**	0	0	0	0	0	**0**
**Total**	**8**	**10**	**23**	**37**	**3**	**81**
