# Supplementary material for: Reproductive Cycle of the Seagrass Zostera noltei in the Ria de Aveiro Lagoon
Source: Plants (Basel). 2021 Oct 26;10(11):2286. doi: 10.3390/plants10112286 (PMC8621667; doi:10.3390/plants10112286)
Supplement: Supplementary file 1 [file plants-10-02286-s001.zip › Table S3.pdf]

Table S3. Seawater temperatures (mean ± SE) among the four studied *Z. noltei* meadows before (May), during (August) and after (December) sexual reproduction period.

**Meadow**	**Before**	**During**	**After**
M1	27.91 ± 0.20	25.15 ± 0.05	13.40 ± 0.01
M2	27.91 ± 0.05	24.75 ± 0.60	13.60 ± 0.10
M3	28.09 ± 0.35	25.22 ± 0.10	13.65 ± 0.05
M4	27.82 ± 0.05	25.78 ± 1.50	14.35 ± 0.15
Average	27.94 ± 0.65	25.24 ± 0.85	13.75 ± 0.14
