# Supplementary figures and images for: Reproductive Cycle of the Seagrass Zostera noltei in the Ria de Aveiro Lagoon
Source: Plants (Basel). 2021 Oct 26;10(11):2286. doi: 10.3390/plants10112286 (PMC8621667; doi:10.3390/plants10112286)

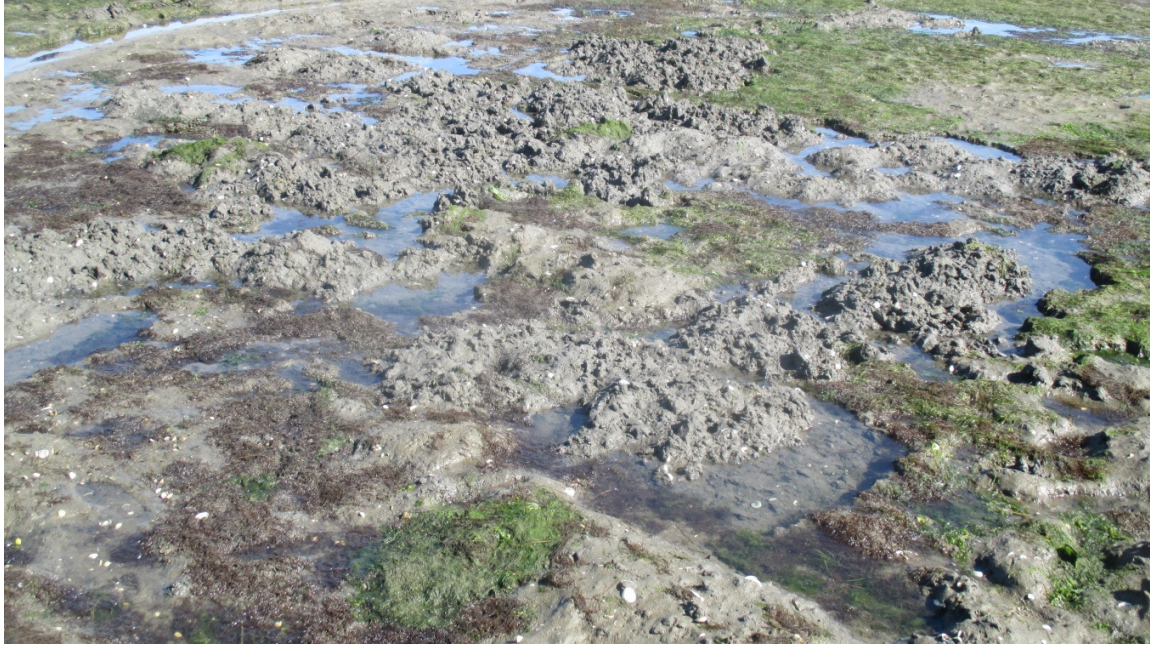

**Figure S1.** Area impacted by shellfish and bait harvesting in the Mira channel of the Ria de Aveiro.

Supplement: Supplementary file 1 [file plants-10-02286-s001.zip › Figure S1.pdf]
